# Effect of Skimmed Milk on the Absorption and Metabolism of5-Fluorouracil (5-FU) in Animals

**Published:** 2012

**Authors:** Maqsood Ahmad, Shazia Akram Ghumman, Alamgeer Sher, Tanveer Sharif, Muhammad Sher, Tahir Abbas

**Affiliations:** a *Department of Pharmacy University of Sargodha, Sargodha, Pakistan. *; b *College of Pharmacy GC University Faisalabad. *; c *Department of Zoology University of Punjab, Pakistan.*

**Keywords:** 5-FU; 5-Fdurd, Plasma concentration, Hematological parameter, Skimmed milk, Route related toxicity

## Abstract

Several modalities of drug administration have been investigated to improve bioavailability and to reduce 5-FU related toxicity. The aim of present study was to evaluate the effect of skimmed milk on the absorption and metabolism of 5-FU in rabbits, mice and dogs. It was further aimed to determine its route related toxicity in rabbits.

Plasma concentration of both 5-FU and its metabolite 5-Fluoro-2-deoxyuridine (5-Fdurd) was determined using HPLC. The absorption of 5-FU co-administered with skimmed milk was significantly higher as compared to its co-administration with water in rabbits and mice (p < 0.001), whereas no significant difference was observed in dogs. The plasma concentration of 5-Fdurd a major metabolite of 5-FU was significantly higher in water group when compared with skimmed milk group in rabbits and mice (p < 0.05), whereas no significant difference was observed in dogs. Route related toxicity was also determined in rabbits. Various hematological parameters were studied at 4^th^ and 7^th^ day after oral and intravenous administration of 5-FU. WBCs count was significantly decreased in intravenous group as compared to control and oral groups (p < 0.001). It was concluded that co-administration of skimmed milk with 5-FU increases its absorption and reduces its metabolism. Skimmed milk also reduces 5-FU related toxicity in rabbits.

## Introduction

5-FU is one of the most commonly prescribed drug for the treatment of solid tumors of breast, GIT, head and neck (1). It is given both intravenously and orally. After IV administration its plasma concentration rapidly fall due to short plasma half life (6 to 20 min) (2, 3). Oral administration of 5-FU is associated with erratic and unpredictable plasma levels with inter and intrapatient variability (4). Attempts have been made to increase the oral absorption and to cope with the problem of pharmacokinetics. 5-FU has been administered: (a) 5-FU prodrug (Capecitabine), (b) 5-FU + DPD inhibitor (5FU/Eniluracil), (c) Ftorafur + DPD inhibitor (UFT) and (d) S-1 combination( Tegafur + oxonic acid + DPD inhibitor) (5). 

Dihydropyrimidine dehydrogenase (DPD), is a major enzyme responsible for the degradation of 5-FU (6). Administration of skimmed milk, a natural antacid has been reported to significantly reduced the susceptibility to the lethal toxicity of 5-FU in mice (7). Exposure of oral mucosa to milk reduces chemotherapy-induced mucositis in Hamster (8). In cancer patients lactose improves the alimentary status and decreases the incidence of leucopenia and thrombocytopenia (9). Skimmed milk improved the bioavailability of 5–FU in colorectal cancer patients (10). The aim of the present study was to evaluate the effect of skimmed milk on the plasma concentration of 5-FU and its active metabolite 5-Fluoro-2-deoxiuridine (5-Fdurd) in rabbits, mice and dogs. It was further aimed to compare its route related toxicity in rabbits.

**Figure 1:**
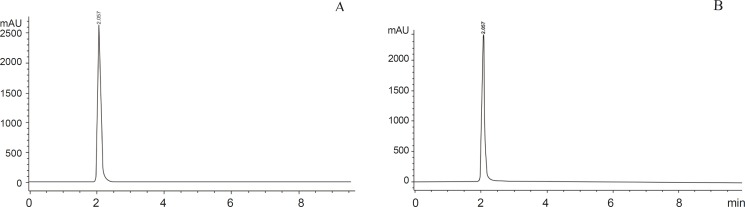
Chromatogram of standard and sample 5-FU. A: standard 5-FU. B: sample 5-FU of rabbit

## Experimental

Ethyl acetate, potassium dihydrogen phosphate, diethyl ether (Sigma Aldrich), n-propanol , ammonium sulphate (Merck) , potassium phosphate (MP Biomedicals) and Double Distilled Water was used. 5-Fluoro-2-deoxyuridine was a kind gift from Ribio Pharmaceuticals, China. *Animals *

Rabbits (Oryctolagus cuniculus) with average weight (1.75 Kg), Mice (Mus musculus Albino mice) with average weight (.037 Kg) and Dogs (German shepherd) with average weight (15.5 Kg) were used and kept in the animal house of University of Sargodha under standard laboratory conditions (12 h light/12 h dark cycle at 23**°**C ± 2**°**C relative humidity 55%). All animals were fed with standard feed, water was available *ad libitum*.

*Calibration of the assay procedure*

The concentrations of unknown samples were determined by using calibration curve constructed with five points from (0-20 µg/mL) for both 5-FU and 5-Fdurd.

*Sample preparation for 5-FU*

500 µL of plasma samples were taken in a 15 mL centrifuge tube, 200 µL of phosphate buffer (55 mM KH_2_PO_4_) and 300 µL of DDW were added to each tube to make volume up to 1 mL. 7 mL of ethyl acetate was added in each tube. Tubes were sealed with rubber stopper and para film. The samples were vortexed for 10 min and centrifuged at 4000 rpm. The supernatant (6 mL/sample) was placed in fresh centrifuge tube and evaporated to dryness under a stream of nitrogen. 

*HPLC description for 5-FU*

500 µL of DDW was added and the mixture was shaken until the residues were completely dissolved. The solubilised sample was filtered through 0.45 micron filter (Millipore) 30 µL of sample was injected onto HPLC system (10). The HPLC system Aglient 1200 model attached with Aglient 1200 Diode Array Detector and Chem Station 32 (Aglient) software was used. The system was adjusted to an absorbency of 260 nm. Separation was accomplished via isocratic elution of mobile phase (50 mM KH_2_PO_4_) with a flow rate of 1 mL/min. A C_18_ Zorbax Eclipse XBD column (5 micron particle size, 4.6+150 mm) was used. HPLC analysis was conducted at 25 **°**C, run time and retention time of 5-FU was 10 min and 2 min respectively (10) (Figure 1).

*Sample preparation for 5-Fdurd*

500 µL of plasma samples were taken in a 15 mL centrifuge tube, 500 µL of saturated ammonium sulphate was added to each tube to make volume to 1 mL. After brief vortex, 4 mL of n-propanol: diethyl ether (80:20 v/ v) were added. After 3 min vortexed samples were centrifuged for 10 min at 2500 g. The organic phase was transferred to a clean tube and evaporated to dryness at 37**°**C under stream of nitrogen. The residue was dissolved in 500 µL of mobile phase and was transferred to an amber glass vial for automatic injection (50 µL) onto the HPLC system. 

**Figure 2:**
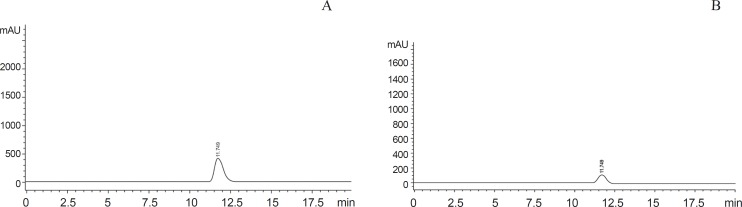
Chromatogram of standard and sample 5-Fdurd. A: standard 5-Fdurd. B: sample 5-Fdurd of rabbit

*HPLC description for 5-Fdurd*

HPLC system described for the determination of 5-FU was used. The samples were run at 210 nm. The mobile phase, contains 1.5 mM K_3_PO_4_ buffer/acetonitrile 99.5:0.5 by volume was adjusted to pH 5 by adding orthophosphoric acid with a flow rate of 1 mL/min. Samples were subjected to HPLC at 25^0^C with run and retention time of 15 min and 11.75 min respectively (17), (Figure.2).

*Determination of plasma concentration of 5-FU after oral administration of 5-FU with skimmed milk and water in Rabbits, Mice and Dogs (n = 5)*

Rabbits were divided into two groups, group I and group II, received 5-FU (20 mg/kg) in 10 mL skimmed milk and water respectively. Mice were also divided into two groups, group III and group IV, received (20 mg/kg) in 0.1 mL skimmed milk and water respectively. Dogs were divided into two groups, groupV and group VI, received (20 mg/kg) in 55 mL skimmed milk and water respectively. Blood samples were taken after 20 min of drug administration in heparinzed tubes. 5-FU was extracted and plasma concentration was determined by using HPLC.

*Determination of plasma concentration of 5-FU metabolite (5-Fdurd) after oral administration of 5-FU with skimmed milk and water in Rabbits, Mice and, Dogs (n = 5)*

Rabbits were divided into two groups, group VII and group VIII, received 5-FU (20 mg/kg) in 10 mL skimmed milk and water respectively. Mice were also divided into two groups, group IX and groupX, received 5-FU (20 mg/kg) in 0.1 mL skimmed milk and water respectively. Dogs were divided into two groups. GroupXI and groupXII, received (20 mg/kg) in 55 mL skimmed milk and water respectively. Blood samples were taken after 20 min of drug administration in heparinized tubes. 5-Fdurd was extracted and its plasma concentration was determined by using HPLC.

*Effect of 5-Fluorouracil on hematological parameters in animal model*

Male albino rabbits belonging to the local strain (Oryctolagus cuniculus) with average weight 1.36 Kg were used. Rabbits were divided into 3 groups (n = 5) in each group. Group1was Control (received no treatment), group 2 Oral (received 20 mg /kg with 10 mL skimmed milk), Group 3 was IV (received 20 mg/kg 5-FU intravenously). Blood samples were taken in heparinized tubes at 4^th^ day and 7^th^ day of drug administration and following hematological parameters were determined. 

1. Red blood cells, white blood cells and platelets counts (RBC, WBC and PLT counts); 2. Hematocrit Percentage (HCT %); 3. Hemoglobin Concentration (HGB); 4. Mean cell hemoglobin (MCH); 5. Mean cell hemoglobin concentration (MCHC).

**Table 1:** Plasma concentration of 5-FU (μg/mL) in rabbits, mice and dogs after oral administration of 5-FU co-administered with skimmed milk & water.

**Groups** **(µg/mL)**	**Plasma concentration of 5-FU (µg/mL)**	**Plasma concentration of 5-FU (μg/mL) **
	**Co-administered with skimmed milk**	**Co-administered with water **
Rabbits	19.38 ± 2.02 **	1.97 ± 1.82
Mice	5.41 ± 0.30 **	0.42 ± 0.27
Dogs	0.15 ± 0.08	0.08 ± 0.01

(Mean ± S.D; n = 5) ** p < 0.001 compared with water.

*Statistical analysis*

Data were expressed as mean ± SD and significance of difference was analyzed by 2-sample t test. Values were considered significant at p < 0.05.

## Results and Discussion

5-FU has remained the most commonly used chemotherapeutic agent in the treatment of advanced colorectal cancer (11). After I.V administration, its plasma concentration rapidly falls due to short plasma half life (6–20) minutes (2, 3). Numerous attempts have been made to increase the therapeutic benefit of 5-FU through schedule modification and biochemical modulation (4, 12). For instance, administration of 5-FU by I.V bolus or continuous infusion, resulted in improved efficacy, but it causes an inconvenience to patient, and often associated with infections and thrombotic complications (13). Biochemical modulation, another strategy to enhance the efficacy of 5-FU, has also resulted in increased toxicity (14). The unpredictable and low oral bioavailability of 5-FU initially makes it unsuitable for oral administration (12). As a result different strategies have been explored in the development of oral preparations, for instance (a) 5-FU prodrugs (b) 5-FU + DPD inhibitor (c) Ftorafur + DPD inhibitor (d) S-1 combination (Tegafur + oxonic acid + DPD inhibitor).These oral preparations caused an increase in anti tumor activity of 5-FU and reduces GI toxicity, CNS toxicity , neutropenia, diarrhea, nausea and alopecia (5). 

Therefore, it was concluded that oral route is more rationale for the administration of 5-FU due to increased preference by the patients and its cost effectiveness (15). Milk enhances the effectiveness of anticancer drug (Baicalein) by thirteen times due to presence of whey proteins in milk (16). Administration of skimmed milk, a natural antacid has been reported to significantly reduced the susceptibility to the lethal toxicity of 5-FU in mice. The increase in the population levels of *E.Coli* in the intestinal tract after administration of 5-FU was inhibited by oral administration of the skimmed milk fraction (7). It was also reported that lactose when given in diet to cancer patients improves the alimentary status and decreases the incidence of leucopenia and thrombocytopenia (9). Exposure of oral mucosa to skimmed milk, pre and concurrent to 5-FU therapy, resulted in significantly reduced mucosal ulceration in Hamsters (8). 

**Table 2:** Plasma concentration of 5-FU metabolite, 5-Fdurd (μg/mL) in rabbits, mice and dogs after oral administration of 5-FU co-administered with skimmed milk & water.

**Groups**	**Plasma concentration of 5-Fdurd (μg/mL) **	**Plasma concentration of 5-Fdurd (μg/mL) **
	**Co-administered with skimmed milk**	**Co-administered with water **
Rabbits	0.15 ± 0.12	3.96 ± 5.21*
Mice	0.11 ± 0.55	0.91 ± 0.84*
Dogs	0.06 ± 0.04	0.15 ± 0.15

(Mean ± SD; n = 5) * p < 0.05 compared with skimmed milk.

*Plasma concentration of 5-FU*

Plasma concentration of orally administered 5-FU with skimmed milk and water was determined in various species. The plasma concentration of 5-FU with skimmed milk was highly significant when compared with water in rabbits and mice (p < 0.001), whereas no significant difference was observed in dogs (Table 1). This increased absorption of 5-FU with skimmed milk is possibly due to whey proteins of milk and it can also be suggested that improved absorption of 5-FU in various species may be due to inhibitory effect of milk towards dihydropyrimidine dehydrogenase enzyme (DPD). Studies over the past two decades have demonstrated that DPD is the initial and important regulatory enzyme in the metabolism of fluoropyrimidine drug 5-FU( 24, 25). 

*Plasma concentration of 5-Fdurd*

5-FU is rapidly metabolized on administration to its active metabolites, (1) 5-fluorouridine (by uridine phosphorylase) and (2) 5-fluoro-2-deoxiuridine (by thymidine phosphorylase) (17, 18). It has been reported that S-1 combination intended to mitigate 5-FU related GI toxicity by preventing the phosphorylation of 5-FU to its active metabolite (5-Fdurd) in the digestive tract (19). Plasma concentration of 5-Fdrud was determine after oral administration of 5-FU with skimmed milk and water in various species to determine the impact of skimmed milk on metabolism of 5-FU to its metabolite 5-Fdurd. Skimmed milk significantly decreases (p < 0.05) the plasma concentration of 5-Fdurd in rabbits and mice as compared to water group (Table 2). This decreased conversion of 5-FU to 5-Fdurd may possibly be due to the inhibition of the phosphorylation of 5-FU by skimmed milk in the digestive tract.

*Variability in absorption*

Several studies have reported inter and intra species variations in bioavailability of 5-FU probably due to a difference in their DPD activity (20, 21). The level of DPD enzyme is varry in different species and within the same species (26). Our results also showed variability in absorption of 5-FU in three different species (rabbits > mice > dogs), that may possibly be due to the variation in DPD activity in different species.

**Table 3:** Various hematological parameters studied at 4th day after 5-FU administration

**Parameters**	**Control**	**Oral with skimmed milk**	**Intravenous **
1. RBCs (10^12^ /L)	5.53 ± 0.60	5.21 ± 0.35	5.41 ± 0.65
2. WBCs (10^9/^/L)	9.76 ± 2.47	8.91 ± 1.41**	5.23 ± 1.32
3. PLTs (10^9/^/L)	30.6 ± 10.87	32.3 ± 11.23	30.25 ± 9.66
4. HCT (%)	33.2 ± 4.24	31.55 ± 3.44	30.85 ± 3.66
5. HGB (g/dl)	11.48 ± 0.99	11.58 ± 2.14	11.35 ± 0.60
6. MCH (pg)	20.86 ± 1.47	22.14 ± 1.98	21.79 ± 2.01
7. MCHC (g/dl)	34.80 ± 2.60	35.43 ± 2.55	36.14 ± 2.98

Values were expressed as (Mean ± SD) ** p < 0.001 compared with intravenous administration.

**Table 4:** Various hematological parameters studied at 7th day after 5-FU administration (n = 5).

**Parameters**	**Control**	**Oral with skimmed mil**	**Intravenous**
1. RBCs (10^12^ /L)	5.53 ± 0.60	5.11 ± 0.48	5.13 ± 0.39
2. WBCs (10^9/^/L)	9.76 ± 2.47	8.76 ± 1.14**	4.88 ± 0.67
3. PLTs (10^9/^/L)	30.6 ± 10.87	31.8 ± 13.10	30.5 ± 9.21
4. HCT (%)	33.2 ± 4.24	32.1 ± 2.84	30.5 ± 2.26
5. HGB (g/dl)	11.48 ± 0.99	11.72 ± 1.24	11.40 ± 0.40
6. MCH (pg)	20.86 ± 1.47	23.23 ± 1.79	21.68 ± 2.11
7. MCHC (g/dl)	34.80 ± 2.60	36.56 ± 2.40	37.12 ± 3.05

Values were expressed as (Mean ± SD) ** p < 0.001 compared with intravenous administration.

*Hematological parameters*

 Intravenous administration of 5-FU causes Septicemia in colorectal cancer patients that may lead to death (22, 23). Septicemia may possibly be due to decrease in WBCs count in these patients. Therefore, leukocyte count at different time intervals is very important to determine the acute toxicity of 5-FU. Route related toxicity of 5-FU in rabbits was also determined by comparing orally administered 5-FU with its IV route. Highly significant difference was noted between control and intravenous group with (p < 0.001). Highly significant difference was also noted between oral and intravenous group with (p < 0.001), (Table 3, Table 4). These findings enable us to conclude that skimmed milk protect the colorectal cancer patients from life threatening toxicity such as Septicemia.

One of the most common and important side effect of 5-FU is Mucositis with ulceration in the oral cavity (27). Intravenous administration of 5-FU also causes Mucositis due to low WBCs count (8). Our results show that WBCs count is improved after oral administration of 5-FU with skimmed milk so patients can be saved from Mucositis.

## Conclusions

The results of this study indicate that (1). It is concluded that skimmed milk tends to increase the absorption of 5-FU in all species under investigation when compared with water, increased absorption of 5-FU may possibly be due to its decreased metabolism into its metabolite 5-Fdurd. 

(2) Variability of absorption in three different species was observed, the order of absorption of 5-FU in three species was Rabbit > mice > dogs.

(3) Skimmed milk also reduces the 5-FU related toxicity. 5-FU is modestly effective in the treatment of solid tumors which can be administered orally with skimmed milk to improve anti tumor activity and to decrease its toxicity (4). Oral administration of 5-FU with skimmed milk improves the WBCs count and protect the patients from Septicemia and Mucositis. 
